# Current status and direct medical cost of amyotrophic lateral sclerosis in the region of Catalonia: A population-based analysis

**DOI:** 10.1371/journal.pone.0223772

**Published:** 2019-10-11

**Authors:** Josep Darbà

**Affiliations:** Universitat de Barcelona, Barcelona, Spain; University of Catania, ITALY

## Abstract

**Introduction:**

Amyotrophic lateral sclerosis is a neurodegenerative disease that leads to motor weakness. There is no cure, and treatment focuses on slowing down progression, which is achieved by a multidisciplinary approach. Hence, it is vital to understand the population needs for an optimal management of the disease.

**Objectives:**

To evaluate the current status of amyotrophic lateral sclerosis in the region of Catalonia, how the disease is managed and its direct medical costs.

**Methods:**

Records corresponding to 841 patients diagnosed between the year 2007 and 2017 were analysed in a retrospective population-based study, including data from primary care centres, hospitals (inpatient and outpatient care), extended care facilities and mental health centres.

**Results:**

Mean diagnosis age was 66.11 years (SD = 12.61) and 52.79% of admitted patients were males. On average, 14.91 months elapsed between diagnosis and death, and the mean age of death was 72.64 years (SD = 12.00). Patients were admitted 10.70 times per year, mostly into primary care (86.50%), although most expenses were concentrated in hospital inpatient care. The mean cost per patient per year was €1,168. The 83.24% of patients had more than 4 systems affected by chronic conditions.

**Conclusions:**

Primary care is of utmost importance in ALS attention in Catalonia, which may have a direct impact in reducing hospitalisation costs. Nonetheless, the expenses linked to inpatient care represent the biggest portion of total costs. Patients’ healthcare usage patterns and the high proportion of patients with multiple chronic conditions should be taken into account in order to adapt and improve guidelines and healthcare systems.

## Introduction

Amyotrophic lateral sclerosis (ALS), also known as motor neurone disease, is a neurodegenerative disease characterised by the degeneration of both lower and upper motor neurons leading to motor weakness that progressively affects patients’ capacity to speak, swallow and breathe [1]. ALS cause is genetic in 10% of cases; in most cases, its etiology remains unknown [2]. Distinct clinical phenotypes have been documented pointing to distinct pathogenic mechanisms [3]. Thus, diagnosis can be challenging and diagnostic delays are not rare. Current estimations situate ALS incidence in Europe at 2.3 per 100,000 males per year and 1.3 per 100,000 women [4]. In Spain, studies based on hospitalisation registries estimate a minimum prevalence of 2 per 100,000 inhabitants [5]. Currently, there is no cure for ALS, and treatment is mostly focused on slowing progression in an effort to improve patients’ life quality. With this aim, a multidisciplinary approach is usually performed, reducing the number of hospitalisations [6, 7]. Hence, an optimal management of the disease should address the aforementioned issues focusing on the population needs.

The evaluation of patient demographics has been proven to be useful in determining the specific needs of patients and physicians regarding the use of healthcare resources, to adapt disease management to disease complexity [8]. To facilitate such evaluations, detailed information on healthcare usage is collected and analysed. In Catalonia, the program for data analysis for research and innovation in health (Programa d'analítica de dades per a la recerca i la innovació en salut, PADRIS) has been developed. This is a specialised linkage program aimed to connect various administrative records from the same patient. PADRIS represents a useful tool for the examination of disease management in order to develop new protocols that reflect the disease complexity within the population [9].

In this context, the objective of this study was to revise the situation of ALS in Catalonia, focusing on patients’ characteristics, disease management and direct medical cost in terms of use of healthcare resources between the years 2007 and 2017.

## Methods

Records of all patients diagnosed with ALS in the region of Catalonia (7.5 million inhabitants) between 2007 and 2017 were extracted from PADRIS via ethics committee approval. Parameters such as health centres and medical history identifiers were re-coded prior to extraction to maintain records anonymised, with no access to identifying information, in accordance with the principles of Good Clinical Practice and the Declaration of Helsinki. The data obtained included detailed information of patients’ use of healthcare resources, comprising 429 primary care centres, 69 hospitals (inpatient and outpatient care), 76 emergency care units (ER), 167 extended care facilities and 5 mental health centres. In all facilities, admission registry included both inpatient and outpatient consultations. Patients’ decease was ascertained using insurance registries (public and private), and was only available between the years 2010 and 2017. ALS patients were ascertained using the 9th revision of the International Statistical Classification of Diseases and Related Health Problems (ICD-9) 335.20 code. Data was provided separately and not all databases contained data for the period of study in its totality; yet, the Health Evaluation and Quality Agency of Catalonia assures data consistency and reliability.

When necessary, the extraction of single-patient information was carried out conserving the first admission of each patient to extract population descriptive statistics.

Direct medical costs associated with the use of healthcare resources were calculated based on the mean cost of admissions and medical procedures determined by the Catalan government for the year 2013 [9, 10]. These figures include all expenses related to the admission: treatment (examination, medication and palliative/surgical care), nutrition, costs associated to personnel, medical equipment and resources. This project was approved by the University of Barcelona's Bioethics Commission.

Subsequently, patient classification into adjusted morbidity groups (GMA), done automatically according to patients’ chronic diseases and into 5 levels of complexity or risk based on data from the year 2016, was used as additive information to disease cost. The Catalan Institute of Health correlates GMA level with a higher number of healthcare admissions of these patients and major medical costs, and risk level 5 corresponds to the 1% of the total population with the highest hospitalisation risk [11]. This correlation provided qualitative complementary data on ALS medical costs.

Data presentation is mainly descriptive. Statistical analyses were performed using Microsoft Excel© Professional Plus 2010 (Microsoft Corporation, Redmond, WA, USA).

## Results

### Patient characteristics

A total of 841 patients diagnosed with ALS between the years 2007 and 2017 were recorded in the database; 444 (52.79%) of the patients were males, 384 (45.66%) were females and in 13 (1.55%) of the files sex information was missing. Patients’ age at the time of diagnosis was 66.11 ± 12.61 years.

Given the disease characteristics, an analysis of patients deceased during the study period was considered of interest. 716 patients with ALS died in this time period, which equals 89.5 patients per year (Table 1). The mean age at death was 72.64 ± 12.00 years. On average, 14.91 ± 1.42 months elapsed between diagnosis and death.

**Table 1:** Patients with amyotrophic lateral sclerosis deceased during the study period (2007–2017). CI: confidence interval.

Number of deceased patients	716
*male patients*	50.28%
*female patients*	44.55%
Deceased per year	89.5 patients
Patients’ age (years)	
*Mean ± SD*	72.64 ± 12.00
Time elapsed (months)	
*Mean ± 95% CI*	14.91 ± 1.42
*Median*	9

### Disease management and costs

**Fig 1:**
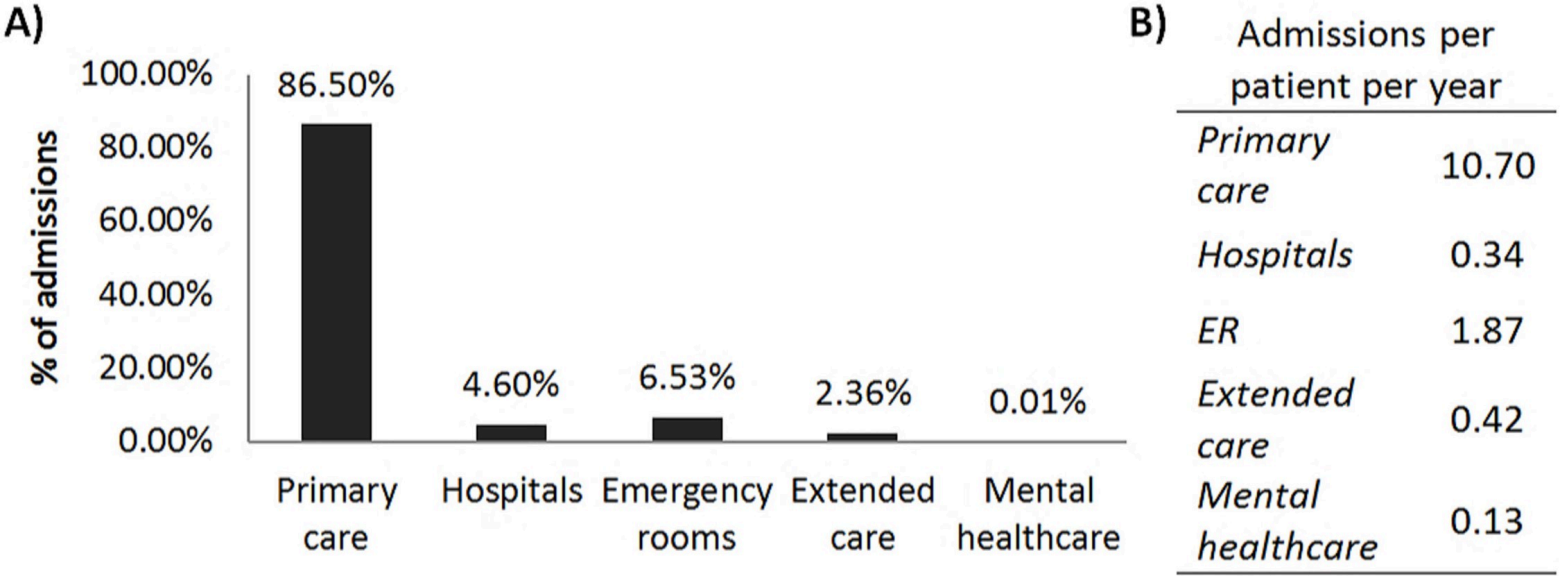
A) Distribution of admissions in the distinct healthcare centres. B) Number of admissions per patient per year.

The aforementioned record files corresponding to the studied population registered 58,775 admissions between 2007 and 2017 in the distinct healthcare centres (primary care, hospitals, ER, extended care facilities and mental healthcare centres) (Fig 1). The 86.50% of all admissions were registered into primary care centres. These centres received on average 10.70 annual consultations per patient. Mean hospitalisation time was 5.63 ± 7.33 days, while time on extended care facilities was on average 107 days, with the median at 18 days. Admissions into ER lasted on average less than a day, with a median of 0 days.

Admissions were classified according to their urgency, with similar proportions of scheduled and urgent consultations in both primary care centres and hospitals (Table 2). All admissions into extended care facilities were considered scheduled. When specified, ER admissions received a high number of patients having minor emergencies or in urgent situations with no vital risk associated. After discharge, most patients moved to their residences, or when discharged from ER, presumably at advanced stages of the disease, 28.71% of patients were transferred to extended care facilities or nursing homes (Table 3). In general terms, data suggested that patient’s death does not occur during hospitalisation events, and only 33.81% of the patients in extended care facilities died during the admission.

**Table 2:** Percentage of patients admitted for scheduled and urgent situations related to ALS in hospitals, ER and primary care.

Origin of admissions	Hospitals	ER	Primary care
Scheduled	48.11%	-	46.01%
Non-scheduled/Urgent	51.89%	-	53,99%
*Non-urgent situations*	-	5.35%	-
*Minor emergencies*	-	15.23%	-
*Urgent situations*, *no vital risk*	-	27.14%	-
*Very urgent situations*, *vital risk*	-	5.81%	-
*Immediate vital risk*	-	0.23%	-
*Unspecified*	-	46.23%	-

**Table 3:** Percentage of patients discharged, transferred or deceased in hospitals, ER and extended care facilities.

Destination after discharge	Hospitals	ER	Extended care
Patients’ residence	82.14%	49.49%	36.55%
Admission for inpatient care	1.17%	16.75%	-
Discharge, external care	4.17%	-	9.34%
Transfer to a extended care facility	3.20%	28.71%	16.24%
Death during admission	8.11%	1.84%	33.81%
Others	1.21%	3.21%	4.06%

Patients with ALS displayed a variety of disease comorbidities. Secondary diagnoses registered in more than 1% of admissions were: hypertension, registered in 3.59% of admissions at some point in patients’ disease course; breathing abnormalities (Including unspecified breath difficulties, distress and insufficiency) registered in 1.41% of admissions; type II diabetes appeared in 1.22% of admissions; acute bronchitis in the 1.15% and disorders of lipoid metabolism, hyperlipidaemia and hypercholesterolemia in the 1.16%, 1.11% and 1.04% respectively.

Most common procedures were: related to facilitating respiration (12%) with measures like mechanical ventilation or oxygen enrichment; imaging techniques (11.32%), including magnetic resonance imaging of the brain and the spinal cord, computed tomography scan of head and abdomen, thorax radiography and thorax tomography. Gastrostomies were performed in 6.34% of the cases, and electromyographies in the 3.05%. Primary care consultations were mainly to receive intramuscular medication, blood test or sanitary education including information on the disease course and managing of the medication.

**Fig 2:**
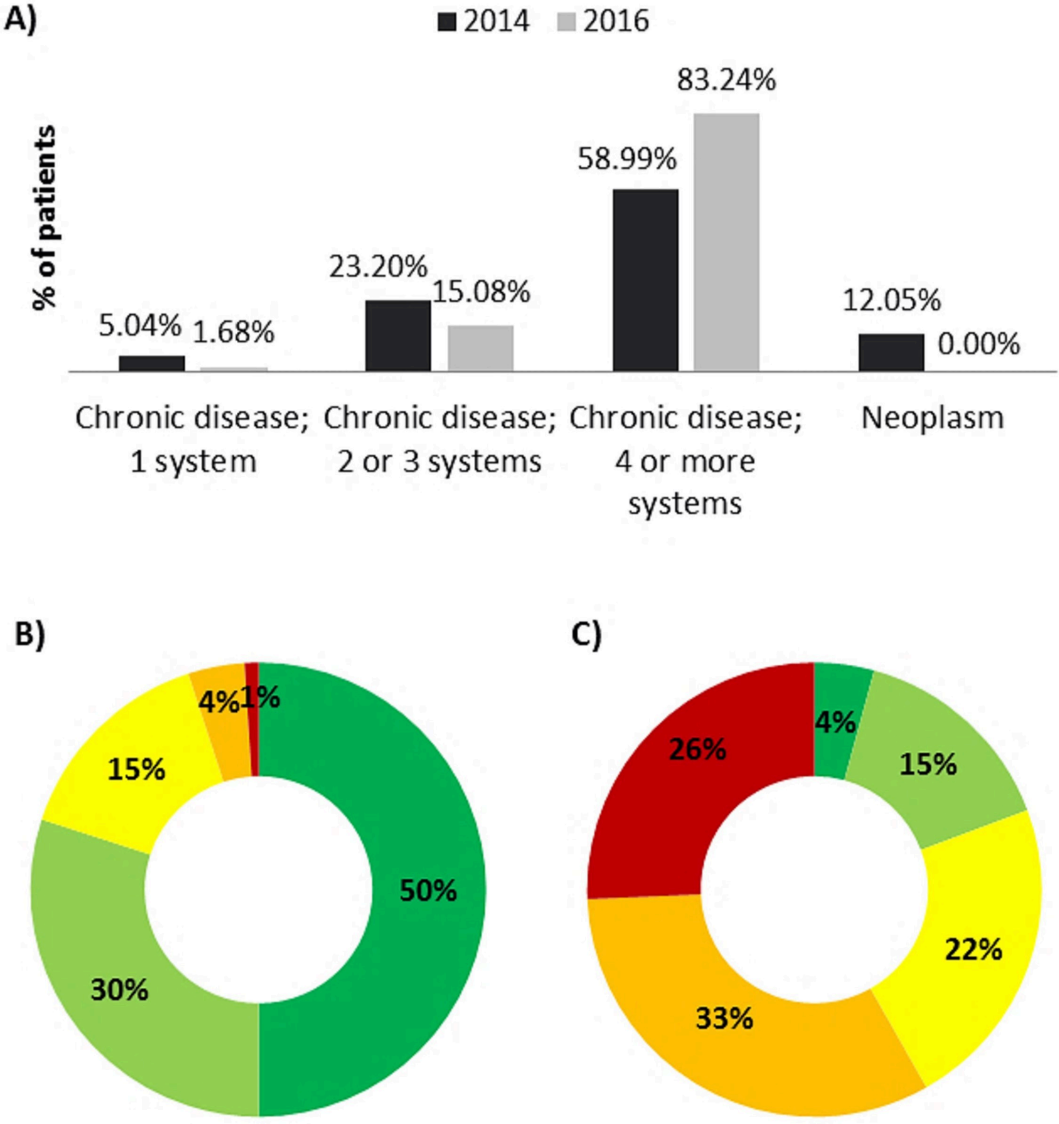
A) Multimorbidities in patients with amyotrophic lateral sclerosis (ALS). Disease complexity levels associated to risk (1 -green- low risk to 5 -red- very high risk) in B) general population [9] and C) patients with ALS in 2016.

Regarding patients’ complexity level and medical costs of the disease, the year 2016 83.24% of patients with ALS had 4 or more systems affected by a chronic condition, 24% more than in 2014 (Fig 2A). In 2016, 26% of patients with ALS were labelled as at a very high risk, and there were almost two times more patients displaying a certain level of risk compared to the general population (Fig 2B and 2C).

On the other hand, the direct cost measurement associated to the use of healthcare resources determined that €7,919,630 were destined to attend patients with ALS between 2007 and 2017 (Table 4). Data was however incomplete due to the characteristics of the database. Taking into account all factors, the cost per year was estimated to be €981,978 with a cost per patient per year of €1,168.

**Table 4:** Direct medical costs associated to patients’ use of healthcare resources (2007–2017) based on mean costs established by the regional government.

	Admissions					Total cost
		*Scheduled*	*Cost*	*Urgent*	*Cost*	
**Primary care**	50,842	22,355	€41	26,230	€62	€2,542,815
		*Mean days of stay*	*Cost per day*	*Cost per admission*		
**Hospitals *Inpatient***	2,319	6.6	€310	€2,046		€4,744,674
		*Cost per admission*				
**Hospitals *Outpatient***	384	€104				€39,936
		*Cost per admission*				
**Extended care**	1387	€67				€93,525
		*Cost per admission*				
**ER**	3836	€130				€498,680
**Total**						**€7,919,630**

Finally, records showed that 88.94% of the patients contributed to only 10% of their pharmaceutical expenses, which were mostly covered by the healthcare system.

## Discussion

### Patient characteristics

The analysis of records of ALS diagnosed patients in Catalonia allowed the assessment of ALS current status in the region, including the evaluation of patient demographics and disease management. In total, 841 patients were included in the study; all diagnosed with ALS between the years 2007 and 2017. Mean age of diagnosis was consistent with previous findings in the north of Spain and worldwide [12, 13]. Generally, incidence rates increase with age, peaking between 65 and 74 and decline after [4].

Regarding disease duration, time elapsed between onset and decease was established at 14.91 months (1.22 years), which is comparable to previous estimations of survival averaging 20 to 48 months; while longer survivals have been measured in patients with early onsets [14].

### Disease management and costs

The Spanish healthcare system, as many others, promotes the use of primary care as a way to maximise healthcare resources [15]. Primary care centres received 86.50% of registered admissions, with similar proportions of scheduled and urgent consultations. Each patient underwent an average of 10.70 consultations each year. ALS management should ensure multidisciplinary care, considering the wide variety of symptoms that impact patients’ life quality [7, 16]. Current treatment guidelines in Spain were published the year 2009, adapting the 2005 European guidelines [17, 18]. Yet, this text left numerous decisions regarding disease management open to interpretation at the healthcare centre level. Posteriorly, diagnosis and treatment guidelines were updated in Catalonia the year 2011 by the Catalan Society of Neurology, emphasising the need for multidisciplinary care explicitly to optimise use of resources and improve life quality and survival [19]. Additionally palliative care and end of life support is considered extremely relevant in these patients [16, 19]. Herein, data suggested that patient’s death occurred principally in extended care facilities or at the patients’ residence.

Regarding disease comorbidities, the system indicated a high proportion of patients with various chronic conditions affecting more than 4 systems (83.24%), presumably corresponding to a great variety of syndromes. This proportion was 3.54 times higher than in the general population, which was 23.5% the same year [20]. Moreover, the Catalan Institute of Health has measured a correlation between the presence of multiple chronic conditions and disease complexity, which is associated with a more intensive use of healthcare [9]. Some of these conditions might correspond to the most frequent secondary diagnoses that included hypertension, type II diabetes, hypercholesterolemia, disorders of lipoid metabolism and breathing difficulties likely to be directly derived from ALS. A previous study linked ALS with several preceding diseases as asthma or diabetes [21], although herein comorbidities cannot be interpreted as precedent diseases. Respiratory difficulties were presumably a consequence of ALS, being a common reason for admission in these patients [22, 23]. Additionally, patients’ old age cannot be ruled out as the cause for this prevalence of chronic diseases. In addition, the database reflects a number of patients at a certain level of risk that doubles that in the general population [9]. These parameters are likely to impact disease burden, increasing medical expenses.

On the other hand, direct medical costs per patient averaged €1,168 per year. Previous estimations in Spain measured a cost per patient of around €1,247 when only hospital admissions, procedures, emergencies and outpatient care were considered [5].

The progressive neurodegenerative nature of ALS determines a more intensive use of healthcare resources. In earlier estimations, when direct medical costs were added to at-home care, follow-up medication, rehabilitation, orthopaedic devices and transportation the sum reached €8,892 per patient [5]. These elements should be included in prospective studies to provide a wider picture of ALS burden. Herein, the database only allowed calculating costs directly derived from admissions, which limited the impact of this study. This factor must be taken into account in prospective resource allocation evaluations. Another limitation was the incompleteness of data due to the database characteristics. Additionally, missing data hampered more consistent analyses.

## Conclusions

The 58,775 admission records analysed revealed the importance of primary healthcare for ALS patients while, hospitalisation costs remain determinant in the calculation of the total burden ALS supposes for healthcare systems. The high proportion of patients with chronic conditions affecting more than 4 organ systems should be taken into account for further research in order to adapt and improve treatment protocols.

## Abbreviations

ALS: Amyotrophic Lateral Sclerosis
ER: Emergency Room
GMA: Adjusted Morbidity Groups
ICD9: 9th revision of the International Statistical Classification of Diseases and Related Health Problems
ICS: Catalan Institute of health (Institut Català de la Salut)
PADRIS: Program of data analysis for research and innovation in health (Programa d’Analítica de Dades per a la Recerca i la Innovació en Salut)
